# Brain lipidomics: From functional landscape to clinical significance

**DOI:** 10.1126/sciadv.adc9317

**Published:** 2022-09-16

**Authors:** Jong Hyuk Yoon, Youngsuk Seo, Yeon Suk Jo, Seulah Lee, Eunji Cho, Amaury Cazenave-Gassiot, Yong-Seung Shin, Myeong Hee Moon, Hyun Joo An, Markus R. Wenk, Pann-Ghill Suh

**Affiliations:** ^1^ Neurodegenerative Diseases Research Group, Korea Brain Research Institute, Daegu 41062, Republic of Korea.; ^2^ Department of Brain Sciences, Daegu-Gyeongbuk Institute of Science and Technology (DGIST), Daegu 42988, Republic of Korea.; ^3^ Department of Biochemistry, Yong Loo Lin School of Medicine, National University of Singapore, Singapore 119077, Singapore.; ^4^ Singapore Lipidomics Incubator (SLING), Life Sciences Institute, National University of Singapore, Singapore 119077, Singapore.; ^5^ Laboratory Solutions Sales, Agilent Technologies Korea Ltd., Seoul, 06621, Republic of Korea.; ^6^ Department of Chemistry, Yonsei University, Seoul 03722, Republic of Korea.; ^7^ Graduate School of Analytical Science and Technology, Chungnam National University, Daejeon 34134, Republic of Korea.; ^8^ Korea Brain Research Institute, Daegu 41062, Republic of Korea.

## Abstract

Lipids are crucial components of cellular function owing to their role in membrane formation, intercellular signaling, energy storage, and homeostasis maintenance. In the brain, lipid dysregulations have been associated with the etiology and progression of neurodegeneration and other neurological pathologies. Hence, brain lipids are emerging as important potential targets for the early diagnosis and prognosis of neurological diseases. This review aims to highlight the significance and usefulness of lipidomics in diagnosing and treating brain diseases. We explored lipid alterations associated with brain diseases, paying attention to organ-specific characteristics and the functions of brain lipids. As the recent advances in brain lipidomics would have been impossible without advances in analytical techniques, we provide up-to-date information on mass spectrometric approaches and integrative analysis with other omic approaches. Last, we present the potential applications of lipidomics combined with artificial intelligence techniques and interdisciplinary collaborative research for treating brain diseases with clinical heterogeneities.

## Teaser

This review article explores lipid alterations in brain diseases and provides insight for future brain lipidomic research.

## INTRODUCTION

With an increase in the aging population worldwide, brain diseases such as neurodegenerative diseases, psychiatric diseases, and brain tumors are being increasingly recognized as leading causes of morbidity and death (*1*, *2*). Dementia has been reported in 50 million people worldwide since 2015, of which approximately 30 million have Alzheimer’s disease (AD) (*3*, *4*). Governments have faced an increased demand for the diagnoses, treatment, and management of patients with brain diseases, making it a national burden in many countries (*1*). In particular, most neurodegenerative diseases are still diagnosed based on clinical symptoms (e.g., cognitive decline, motor impairment, and communication difficulties) and their pathological mechanisms are unclear, thereby limiting the number of treatment approaches available (*5*, *6*). Brain diseases often exhibit a variety of molecular phenotypes and progression patterns, driven by complex genetic, biological, and environmental factors (*7*, *8*). Hence, deciphering the molecular pathways and networks in the brain could lead to the characterization of the pathology of brain diseases and the design of appropriate therapeutic strategies.

Initially, molecular analyses of brain diseases were conducted using a small subset of components associated with specific pathways (*9*, *10*). However, these approaches cannot adequately decipher the concurrent molecular aberrations occurring during disease development. Recently, integrative analyses of omics, such as genomics, transcriptomics, proteomics, and metabolomics, have been used to elucidate systematic and multifactorial molecular alterations in the brain (*8*, *11*). Genomic, transcriptomic, and proteomic analyses of brain diseases have been extensively explored using well-established analytical and bioinformatic platforms (*12*–*14*). Genome-wide association studies (GWAS) and meta-analyses of GWAS have allowed genomic investigation on a large scale, encompassing of the entire genome, thereby enabling the identification of genetic variants and disease risk loci (*12*). Furthermore, next-generation sequencing (NGS) techniques, whole-exome sequencing, and whole-genome sequencing are very powerful and useful tools for detecting previously unknown genes in rare genetic variants undetected by GWAS (*15*). With growing interest in the discovery of the genetic risk loci associated with brain diseases, transcriptomic approaches using microarray-based gene and exon arrays and NGS-based RNA sequencing have been used to further confirm these genetic findings and their interpretations (*16*, *17*). Moreover, brain proteomics has been explored extensively using mass spectrometry (MS)–based platforms and multiplex immunoassays (*13*, *18*). Notably, vast proteomic databases and software tools for data processing of massive MS spectra datasets have contributed to the determination of changes in the proteome, the biological ramifications, and systemic contexts (*14*). Lipidomics has recently been used for the integrative analysis of brain omics, resulting in extensive studies on the association between genetic variations, alterations in protein expression, and lipid metabolism (*16*). However, in lipidomics, there remains the analytical challenge of achieving the inclusion of all lipids present in biological samples, such as cells, tissues, and biofluids, and determining the accurate quantities of numerous, structurally diverse lipids (*19*, *20*). Continued analytical development and database expansion for lipidomics are necessary to elucidate the molecular mechanisms of the lipids in the brain.

Nevertheless, lipidomic analysis is becoming a useful tool in biomedical research. Lipids are essential components of human physiology. They act as key constituents of cellular membranes, affecting membrane synthesis and signal transduction (*21*). A constant, substantial transfer of lipids commonly occurs between cellular membranes. Determining flow patterns of lipids associated with specific cellular mechanisms is an appropriate approach to understanding cellular events (*22*, *23*). Therefore, understanding changes in lipid metabolism and their trafficking has contributed largely to understanding the mechanisms behind various human diseases, including cancer, neurodegenerative diseases, diabetes, and obesity (*24*, *25*). A perturbation in metabolic pathways and reactions, particularly lipid homeostasis, mitochondrial bioenergetics, oxidative stress, inflammation, and neurotransmission, has been observed in brain diseases (*26*). Here, we have reviewed the biological roles of lipids in the physiology of the brain and the role of altered lipid metabolism in brain disease pathology. Last, we have presented applications of brain lipidomics in future clinical research.

## BRAIN LIPIDS

Lipids are common biomolecules and are the primary components of cellular membranes. They are complex metabolites synthesized mainly by enzymes in the endoplasmic reticulum (*27*). In humans, the total number of molecular lipid species is still a matter of discussion but has been estimated at more than 100,000 due to the structural diversity that arises from the combination of diverse head groups, their distinct locations, the unsaturation level of the lipid backbone, and the position of carbon-carbon double bonds (*28*). Despite tremendous molecular complexity, the field of lipidomics has advanced the understanding of lipid metabolism and lipid-associated diseases, leading to the discovery of biomarkers for the diagnosis and prognostic monitoring of diseases (*29*). Lipids in blood plasma and serum have been the primary research targets owing to the ease of samplings. Blood lipidomic studies have enabled the successful detection of metabolic diseases such as diabetes and other systemic diseases such as cancers, infectious diseases, and neurodegenerative diseases (*24*, *30*, *31*). Lipids in the cerebrospinal fluid (CSF), as well as blood, are considered potential markers in patients with AD, Parkinson’s disease (PD), and schizophrenia (SCZ) (*32*–*34*). CSF often contains primary metabolites diffused from the brain, making it a unique sample source that reflects brain disease pathology (*35*, *36*). Lipidomic studies of the brain have been performed, mostly using biofluids such as blood and CSF. However, the available lipidomic information is limited and, thus, insufficient to understand lipid-associated brain homeostasis and elucidate the lipid dysregulation that occurs during brain diseases.

**Fig. 1.**
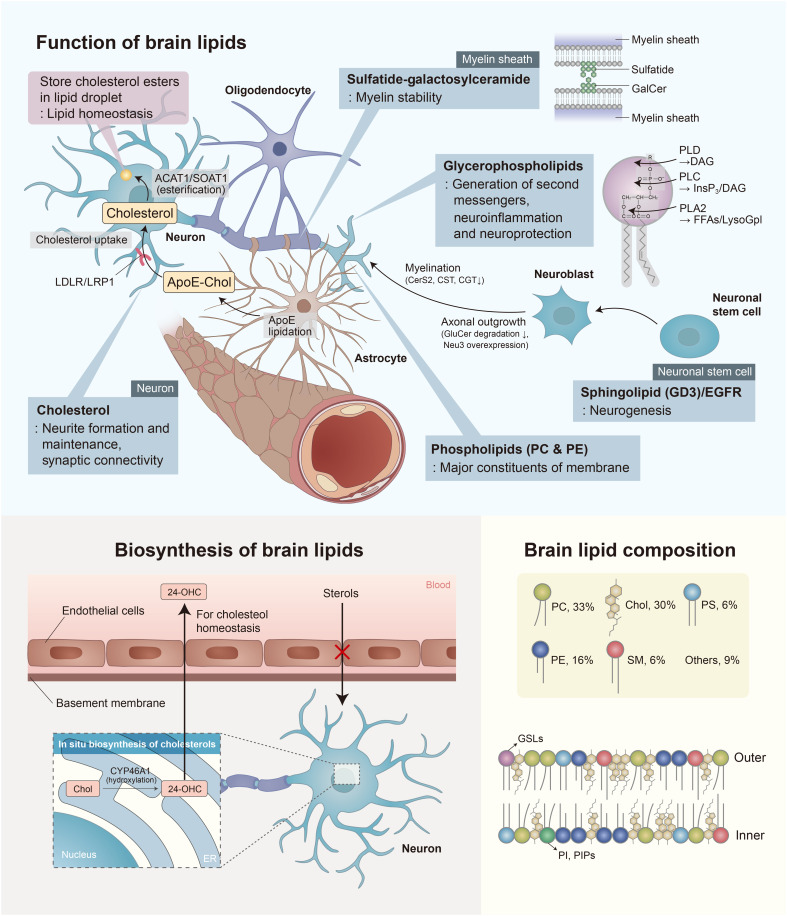
Functions and characteristics of brain lipids. Brain lipids are primarily composed of PC, cholesterol (Chol), PE, phosphatidylserine (PS), and SM, which are involved in brain functions including organ homeostasis, cell formation and maintenance, and signal transduction. Chol is synthesized in astrocytes and transferred to neurons via the formation and secretion of Chol-rich apolipoprotein (APOE-Chol). Certain Chols of neurons are transformed into 24-hydroxycholesterols (24-OHCs), which are then released into bloodstream. The degradation of glycerophospholipids by phospholipase A (PLA), phospholipase C (PLC), and phospholipase D (PLD) leads to the generation of second messengers. Neurodevelopment progresses via the regulation of enzymes associated with lipid synthesis, such as ceramide synthase 2 (CerS2), ceramide galactosyltransferase (CST), ceramide galactosyltransferase (CGT), and *N*-acetyl-α-neuraminidase 3 (Neu3). PIP, phosphatidylinositol-phosphate; GSL, glycosphingolipid; GluCer, glucosylceramide; FFA, free fatty acid.

Of all human tissues, the brain has the second-highest lipid content after adipose tissues, accounting for 50% of its dry weight (*37*). Brain lipids mainly consist of cholesterol, phospholipids, such as phosphatidylcholine (PC) and phosphatidylethanolamine (PE), and sphingolipids (Fig. 1) (*27*). Notably, the brain contains a high level of cholesterol compared to other organs and biological fluids (*27*, *38*). Most sterols in the central nervous system (CNS) are synthesized in situ because the blood-brain barrier (BBB) prevents the transfer of blood sterols into the brain. On the other hand, free cholesterol, mainly 24-hydroxycholesterol, crosses the BBB and leaks into the CNS—a key process in the maintenance of cholesterol homeostasis (Fig. 1) (*39*). The synthesis of phospholipids and sphingolipids is initiated by fatty acids (FAs), which are essential components of most types of lipids (*40*, *41*). The brain mostly produces saturated FAs, while its ability to synthesize polyunsaturated FAs (PUFAs) is relatively poor (*42*, *43*). These PUFAs are mostly obtained from peripheral blood by passive diffusion or by adenosine triphosphate–dependent transporter protein–mediated mechanisms (*40*, *42*). Nevertheless, the transcriptional machinery for the biosynthesis of PUFAs and for long-chain PUFA (LCPUFA)–containing phospholipid remodeling is still present in brain cortex and subjected to dietary [i.e., docosahexaenoic acid (DHA)–poor/rich diets] and hormonal regulation (i.e., estrogens). This is critical for brain lipid homeostasis in periods of low supply of precursors or high external demands (i.e., pregnancy) (*41*). PUFAs such as DHA and arachidonic acid modulate synaptic plasticity and neurotransmission (*44*, *45*). Membrane phospholipids are synthesized from common precursors like diacylglycerol (DAG) and phosphatidic acid (PA), which consist of a glycerol backbone, a saturated FA, and an unsaturated FA (*46*). PC and PE, the most abundant glycerophospholipids in cellular membranes, are synthesized from PA via the Kennedy pathway (*47*). Sphingolipids, on the other hand, consist of a sphingosine backbone linked to a single FA. Ceramides are further modified by branching and by the addition of hydroxyl groups, resulting in the production of sphingomyelin (SM), cerebrosides, and glycosphingolipids (fig. S1) (*48*). FAs in the brain are an important source for the synthesis of phospholipids and sphingolipids, as well as serving as energy substrates and bioactive molecules. The oxidation of FAs, which occurs entirely in astrocytes, accounts for approximately 20% of the total energy needs of the brain (*49*). In the subsections below, we have detailed the biological functions and characteristics of the major lipid molecules in the brain (i.e., cholesterol, glycerophospholipids, and sphingolipids).

### Cholesterol

Brain cholesterol accounts for 25% of the total cholesterol found throughout the body. Cholesterols, which are mostly generated from astrocytes, are transferred to neurons through the formation of cholesterol-rich lipoproteins, including apolipoprotein E (APOE) (*39*). In neurons, cholesterol is used for neurite maintenance and synaptic connectivity (*27*, *39*). In addition, endogenous cholesterol in the brain is primarily found in myelin sheaths (*50*). Unesterified cholesterol occurs at a higher level in the brain compared to that in other tissues. Approximately 1% of the brain cholesterol is esterified and occurs as lipid droplets that store surplus cholesterol inside cells (*50*). The BBB prevents, with very few exceptions, the exchange of sterols between the CNS and peripheral tissues. Consequently, the daily exchange rate between the brain and peripheral is estimated to be lower than 1%, and cholesterol metabolism in the brain can be considered almost independent of peripheral tissues (*42*, *51*). Therefore, normal cholesterol homeostasis is important for maintaining brain function, and its disruption can lead to neurodegenerative diseases and cognitive deficits in the elderly (*52*, *53*).

### Glycerophospholipid

Glycerophospholipids, including PC and PE, are the main phospholipid components of cell membranes (*54*). Alterations in their composition affect the stability, permeability, and fluidity of neural membranes, which, in turn, leads to neurological diseases (*54*). The PC and PE of brain cells, like in other tissues, often control the membrane-anchoring of proteins (*27*, *55*). In addition, the receptor-mediated degradation of glycerophospholipids by phospholipases A, C, and D results in the production of second messengers, such as DAG, inositol 1,4,5-trisphosphate, lysoglycerophospholipid, platelet-activating factor, and LCPUFAs (*54*, *56*). Especially, LCPUFAs are a source for eicosanoids and docosanoids that play important roles in neuroprotection and anti-inflammation in the CNS. (*57*)

### Sphingolipid

Sphingolipids, commonly observed in the nervous system, are essential components of cellular membranes. Hence, they are involved in maintaining brain function, particularly neurogenesis and synaptogenesis (*58*, *59*). Sphingolipids in lipid rafts, together with cholesterol, are associated with the activity of transmembrane proteins (*60*), while the sphingolipids of synaptic membranes interact with neurotransmitter receptors and regulate their activity (*61*).

Sphingolipids include SM, gangliosides, cerebrosides, and sulfatides, all of which are derived from ceramides (*N*-acetylsphingosine) (fig. S1) (*27*, *62*). A high level of SM occurs in the white matter of the brain as the primary component of the myelin membrane (*63*). Cerebrosides are also observed in white matter at a higher level than in gray matter (*27*). Most of the cerebrosides in the brain are galactosylceramides, ceramides with a galactose residue. Glycosynapses, composed of galactosylceramide and sulfatide on opposite membranes of myelin sheaths, are involved in long-term myelin stability (*59*). Last, gangliosides, which are glycosphingolipids with sialic acid residues, are structurally different in terms of monosaccharide content and glycosidic linkages (*64*). Gangliosides are abundant in the CNS, including the brain, and are associated with cell signaling and neuroprotection (*59*). During neuronal stem cell proliferation, the ganglioside GD3 of the cellular microdomain initially colocalizes with the epidermal growth factor receptor (*65*). After that, the axonal outgrowth of neuroblasts can be stimulated via the inhibition of glucosylceramide degradation and the overexpression of *N*-acetyl-α-neuraminidase 3 (Neu3) (*59*).

## LIPIDOMICS IN BRAIN DISEASES

In brain research, lipidomics can be used to discover biomarkers for the early diagnosis and prognosis of brain diseases and to understand the biological functions of the brain and its specific regions (*66*). Various types of lipids and their dysregulation have been implicated in neurodegenerative diseases and mental diseases (*67*–*69*). A summary of the recent studies on the association between lipidomics and brain diseases is provided in Table 1.

**Table 1.** Lipidomics associated with brain diseases. 4-HNE, 4-hydroxynonenal; KO, knockout.

**Brain disease**	**Model**	**Associated lipid species**	**Description**	**Reference**
AD	• Human	Cholesterol and sphingolipid	• In the frontal grey matter of AD patients with *APOE*4	(*74*)
	• Brain tissues		• The increase of cholesterol, cholesterol ester, and ceramide	
	• AD, *n* = 30 (*APOE*3, *n* = 15/*APOE*4, *n* = 15) and Control, *n* = 26 (*APOE*3, *n* = 20/*APOE*4, *n* = 6)		• The elevation of a lysine adduct of 4-HNE	
			• The altered metabolism of sphingolipid and sterol affected on cellular oxidation, increasing the 4-HNE level.	
AD	• Human	Cholesterol and sphingolipid	• In the entorhinal cortex and cerebellar vermis of patients with AD	(*71*)
	• Brain tissues		• (Lipid rafts) low levels of cholesterol and sphingomyelin	
	• AD, *n* = 19/ Control, *n* = 15		• (Lipid rafts) high phosphatidylcholine	
AD	• Human	Sphingolipid	• In the cytosolic fractions of normal and AD brain	(*80*)
	• Brain tissues		• (AD) The increase of ASM and AC	
	• AD, *n* = 9/ Control, *n* = 6		• (AD) The reduction of SM and the increase of ceramide	
AD	• Mouse model	Phospholipid	• (APP and phospholipid transfer protein KO mice) phosphatidylethanolamine and phosphatidylserine were decreased	(*87*)
	• Brain tissues			
	• *APP*, *n* = 5/ *APP*&*PLTP*ko, *n* = 5		• PLTP deficiency can disrupt *APP* maturation or transport.	
PD	• Human	Triacylglycerol	• In the visual cortex of patients with PD	(*69*)
	• Brain tissues			
	• PD, *n* = 10/ Control, *n* = 10		• Decreased TAGs, increased DAGs	
LBD	• Human	Phospholipid-cholesterol	• The increase of phospholipid-cholesterol	(*152*)
	• Brain tissues			
	• LBD, *n* = 8/Control, *n* = 10			
HD	• Human	Sphingolipid	• In the striatal and cortical specimens from HD brain, up-regulated S1P lyase 1 and down-regulated sphingosine kinase 1 were found.	(*103*)
	• Brain tissues			
	• HD, *n* = 3/ Control, *n* = 3		• (R6/2 mouse model overexpressing the exon 1 of the human HD gene) reduced sphingosine- 1-phosphate and increased sphingosine	
HD	• Human	Cholesterol	• In the caudate, putamen, and cerebellum of patients with HD	(*67*)
	• Brain tissues		• Different concentrations of cholesterols between three regions	
	• HD, *n* = 13/ Control, *n* = 13		• (HD caudate and putamen) the elevated CE	
SCZ	• Human	Phospholipid and sphingolipid	• (SCZ) decreased PCs, PEs, and LPCs	(*68*)
	• Serum			
	• SCZ, *n* = 91/ Control, *n* = 109		• (SCZ) increased SMs and LPEs	

### Alzheimer’s disease

AD is the most common neurodegenerative disease and the most common cause of dementia (*51*). It is well known that histopathological changes in AD are associated with amyloid plaques and neurofibrillary tangles. However, the association between the symptoms of AD (e.g., memory loss and cognitive dysfunction) and their causes remains poorly understood (*70*). Studies on lipid alterations are drawing attention to the molecular mechanisms underlying amyloid precursor protein (APP) processing, amyloid beta (Aβ) production, and Aβ aggregation (*71*).

Initial studies linking lipids to AD pathogenesis identified a dysregulation in cholesterol trafficking from astrocytes to neurons. In the brain, APOE is the main cholesterol carrier and is capable of binding and clearing Aβ peptides (*72*). Previous studies have shown that a genetic variant of APOE, encoded by the *APOE*ε*4* allele, is one of the risk factors of AD and is linked to alterations in cholesterol and sphingolipids (*73*, *74*). No direct association has been identified between cholesterol and amyloidogenesis; however, the role of brain cholesterol in APP processing that occurs in lipid rafts has been explored (*71*). Lipid raft regions of the cell membrane are cholesterol and sphingolipid enriched, and these anchor AD-related transmembrane proteins such as β-site APP cleaving enzyme 1 (BACE1) and γ-secretase. Previous research has demonstrated that cholesterol in lipid rafts is involved in reducing the distance between APP and BACE1 before rapid endocytosis (*75*, *76*). In addition, the protein activity of BACE1 and γ-secretase is influenced by cholesterol levels, suggesting the impact of cholesterol metabolism on the occurrence of AD (*77*).

Sphingolipid metabolism in the brain is highly associated with the formation of an Aβ oligomer, Aβ42 (*78*, *79*). Compared to brains of healthy individuals, those of patients with AD show high levels of acid sphingomyelinase and acid ceramidase, which lead to a reduction in the level of SM and production of ceramides (*80*). Accumulation of SM decreases γ-secretase activity, resulting in a reduction in Aβ secretion (*78*, *79*).

Ceramides stabilize BACE1 and increase its half-life, thereby increasing the rate of Aβ formation (*81*). Glycosphingolipids also contribute to the formation of amyloid fibrils (*82*). The glycolipid monosialo-tetrahexosyl-ganglioside (GM1) binds to released Aβ. These GM1-Aβ complexes have been found in the brains of patients with early AD, and the abundance of these complexes has been correlated with the Aβ oligomers observed in the CSF (*83*). Collectively, sphingolipids, essential components of cellular membranes, influence APP processing, Aβ production, and the subsequent event, amyloid aggregation.

Phospholipids are associated with the activities of γ-secretase and proteins involved in APP processing. Previous studies on the brains of patients with AD have reported changes in various types of phospholipids, including PC, PE, and phosphatidylinositol, as well as phospholipid-metabolizing enzymes such as phospholipase C (PLC) and phospholipase D (PLD) (*79*). Phosphatidylinositol-4,5-bisphosphate, a well-characterized derivative of phosphatidylinositol, is a substrate for hydrolysis by PLC. Several studies have shown that PLC inhibition leads to a reduction in phosphatidylinositol-4,5-bisphosphate turnover, thereby leading to a reduction in Aβ42 secretion (*84*). In contrast, blocking the PLC activity has been found to negatively affect the activity of non-amyloidogenic α-secretase (*85*). Therefore, whether PLC signaling is beneficial in suppressing amyloidogenesis in AD is uncertain. Recent studies have revealed alterations in the level of plasmalogen PE (PE-P) found in the white and gray matter in the brains of patients with AD (*86*). The level of PE-P in the white matter of the brain declines by approximately 40% in early AD, whereas that in the gray matter increases from 10 to 30% with disease progression (*86*). This finding suggests that PE-P could be a potential lipid biomarker for AD. With respect to the cognitive impairment associated with AD, the deficiency of the phospholipid transfer protein, which induces a decrease in PE and phosphatidylserine, accelerates the intracellular accumulation of Aβ and memory dysfunction, indicating a correlation between phospholipid metabolism and APP processing (*87*). In addition, it was demonstrated that LCPUFAs (DHA and amino acid) are depleted in mouse and human AD brain membranes (particularly in lipid rafts) and that it affects the neurochemical and physicochemical properties of nerve cell membranes and pro-amyloidogenic processing. This depletion occurs in normal aging (with gender bias) but, when exacerbated, provides a mechanistic link between pathological aging and AD (*88*).

### Parkinson’s disease

PD is the second most prevalent neurodegenerative disease that occurs in people over 60 years of age (*89*). Patients with PD show symptoms involving the loss of motor functions such as tremors, slowness of movement, rigidity, and impaired balance. Besides motor symptoms, cognitive alterations are also common in patients with PD, often leading to dementia (*90*). In the past decades, PD-associated cellular pathways involved in oxidative stress, endosomal-lysosomal dysfunction, endoplasmic reticulum stress, and immune response have been found. However, no cure for PD has been reported (*89*, *91*–*93*).

The hallmark feature of PD is the aggregation of α-synuclein (α-syn), a neuronal protein that affects the regulation of synaptic vesicles and neurotransmitter release, the subsequent production of Lewy bodies (i.e., fibrillized α-syn), and the loss of dopaminergic neurons (*94*). The N-terminal region of α-syn is rich in basic residues, including KTKEGV peptide repeats, indicating a high affinity for lipid membranes (*95*). In familial PD, the N terminus of α-syn has six specific mutations: A30P, A53T, A53E, H50Q, E46K, and G51D (*96*). These mutations appear either together or separately in α-syn, and the type of mutation dictates the differences in the membrane-binding affinity of α-syn (*96*). Previous studies have shown that α-syn has an affinity for negatively charged phospholipids, leading to its accumulation on the phospholipid layer of lipid droplets containing a high level of triacylglycerols (*97*). In contrast, the lipid-binding affinity of mutant α-syn, including A30P, is significantly reduced. Although the A53T mutant of α-syn binds to lipid droplets, it leads to the hydrolysis of the stored triacylglycerols (*97*). The primary visual cortex of patients with PD shows reduced TAG levels (*69*). In addition, a postmortem study of patients with Lewy body dementia (LBD) indicated a reduction in the expression level and activity of PLD1. The membrane-bound lipases, PLD1 and PLD2, are involved in neurotransmitter release. In addition, PLD1 prevents α-syn accumulation and cytotoxicity through the activation of autophagic flux (*90*, *98*). Lipid alterations in membrane microdomains (lipid rafts) have been solidly demonstrated in human frontal cortex from PD donors. This is consistent with the cognitive deficits mentioned before. These alterations are present in patients with incidental PD (milder stage) and also in LBD, a closely related neurological disease (*94*).

### Huntington’s disease

Huntington’s disease (HD) is an inherited progressive brain disease characterized by abnormal motor functions (chorea and dystonia), psychiatric complications (anxiety and depression), and cognitive functions (dementia) (*99*). The mutation in the Huntingtin (*HTT*) gene is a well-known cause of HD (*67*). The *HTT* gene and other associated proteins are involved in intracellular functions, such as postsynaptic signaling, protein trafficking, and protein aggregation (*100*–*102*). Compared to the lipidomic studies on AD and PD, little research has been conducted on lipid alteration in HD.

Di Pardo *et al.* (*103*) have identified differences in levels of sphingosine-1-phosphate (S1P)–metabolizing enzymes among patients with HD and healthy controls. Specifically, the postmortem striatum and cortex of patients with HD, unlike controls, exhibited up-regulated S1P lyase 1 and down-regulated sphingosine kinase 1 level, indicating a perturbation in sphingolipid metabolism in patients with HD (*102*). In addition, human studies on HD have investigated disturbances in the levels of neural cholesterol and cholesteryl ester (CE) (*80*). In particular, the caudate and putamen of patients with HD have been shown to contain elevated levels of CE, which, in turn, reduces cholesterol accumulation as a counteracting mechanism (*67*).

### Schizophrenia

SCZ is a psychiatric disorder in which people show symptoms of disordered thinking and behavior, hallucinations, and delusions. It cannot be sufficiently treated with antipsychotic drugs (*68*, *104*). The neurobiology of SCZ has been explained by changes in the dopaminergic, glutamatergic, and serotoninergic signaling pathways (*105*). Drugs prescribed for SCZ treatment have been used to normalize neurotransmission dysfunctions (*105*). A few studies have revealed that the postmortem cerebral cortical tissues of patients with SCZ contain abnormal compositions of membrane phospholipids (*105*, *106*). These changes in the lipid composition of neuronal cell membranes may affect the storage and release of neurotransmitters (*105*, *107*). Furthermore, analysis of brain tissues and blood samples of patients with SCZ has revealed changes in lipidomes. It was found that 10.4% (525 of 5024) of PFC (prefrontal cortex) lipids of patients with SCZ have significantly altered concentrations compared with healthy individuals (*108*). Changes in these PFC lipids likely increase membrane fluidity compared to age-matched controls and similarly in cognitively healthy individuals of older ages compared to age-matched ones (*108*). Evaluation of serum samples for biomarkers of SCZ has revealed that levels of all PEs, as well as many PCs and lysophosphatidylcholines are lower, while those of SMs and most lysophosphatidylethanolamines are higher in patients with SCZ compared to controls (*68*). Specifically, six lipids, LPC (18:0), LPC (20:2), PC (18:2/18:2), PC (O-16:0/18:2), LPE (20:4), and PE (P-18:0/18:2), were shown to be differentially expressed in patients with SCZ, indicating their potential for use as disease biomarkers.

## CURRENT TRENDS IN BRAIN LIPIDOMICS

### MS-based lipidomics

A variety of techniques have been used for brain lipidomic studies, including nuclear magnetic resonance (NMR) spectrometry, fluorescence assay, and MS (*19*). These techniques exhibit different analytical performances, in terms of sensitivity and efficiency, and the technique of choice depends on the purpose of the study. Fluorescence assays are the simplest technique to quantify specific lipid components; however, they are not suitable for in-depth profiling of lipids (*109*, *110*). Compared to MS-based tools, NMR is less sensitive but has certain advantages such as nondestructive sample preparation and detailed structural elucidation capabilities (*111*, *112*). Recently, techniques such as shotgun MS, liquid chromatography–MS (LC-MS), and gas chromatography–MS (GC-MS) have been used in lipidomic studies owing to improvements in instrumental performance (*108*, *113*). State-of-the-art MS, which exhibits high sensitivity and high resolution, allows in-depth analysis of the compositions and structures of a vast array of lipids including isomers (*114*). Determination of acyl chains *sn*-positions and confirmation of the carbon-carbon double bonds position and conformation are still challenging, but recent advances using techniques such as the Paternò-Büchi reaction, ozonolysis, ultraviolet photodissociation, and electron impact excitation of ions from organics are pushing the boundary of routine lipid characterization (*115*–*120*). In addition to MS systems coupled to chromatographic separation, the MS imaging (MSI) technique using matrix-assisted laser desorption ionization (MALDI) is also frequently used to reveal the regional and spatial distribution of specific brain lipids (*121*, *122*), thereby enabling the in situ elucidation of the molecular-histological map of the brain. For lipid imaging using MALDI-MSI, frozen brain tissue is sectioned into thin slices and covered by a matrix through the process of sublimation (*122*). Subsequently, the prepared brain slice is directly analyzed via MALDI-time-of-flight (TOF) MS or TOF-MS. This enables the spatial identification of the lipid species in the tissue. Recent lipidomic studies have used MALDI-MSI to show that the abundance of some lipid species is altered in AD and to pinpoint the localization of this alteration, specifically to the cornu ammonis 1 region of the hippocampus (*123*). MSI is an efficient tool for spatial analysis of the lipid species in the brain; however, absolute quantification using MSI remains problematic (*122*, *124*).

**Fig. 2.**
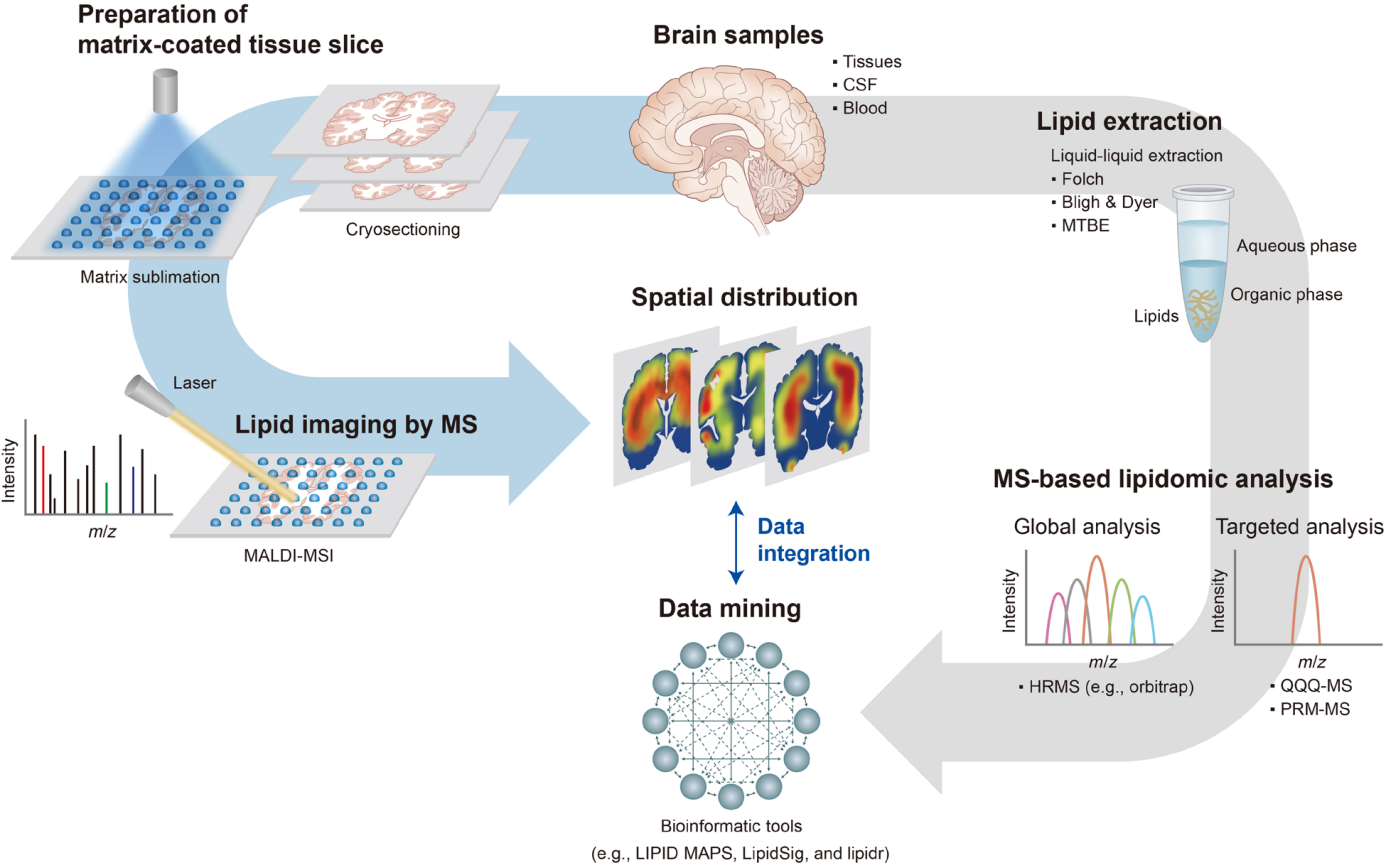
MS-based analytical strategy for brain lipidomics research. Lipidomic analysis of brain tissues and biofluids involves sample treatment, MS analysis, and data processing. The general strategy of lipidomics involves lipid extraction followed by the separation and detection of lipid mixtures using LC-MS (right arrow). Conversely, lipid imaging using MALDI-MSI involves the direct analysis of lipids in brain tissue slices without their extraction. Here, the frozen brain tissue is sectioned and matrix-coated before MS analysis. Following analysis, the MS data of lipids, such as mass value and ion intensity, are converted into an image file (left arrow). *m/z*, mass-to-charge ratio. HRMS, high-resolution mass spectrometry; PRM, parallel reaction monitoring; MTBE, methyl-tert-butyl ether.

Figure 2 represents a streamlined workflow for MS-based studies of brain lipidomics. Lipids in brain tissues, cell pellets, and biofluids such as serum, plasma, and CSF are obtained using liquid-liquid extraction methods, such as Folch, Bligh, and Dyer, and methyl tert-butyl ether or *n*-butanol/methanol methods (*125*, *126*). Typically, for the MS-based quantitation of lipids with different structures, internal standards of diverse lipid classes are added to the sample before extraction. Optionally, the whole lipid mixture is fractionated into polar and nonpolar components to reduce the molecular complexity and to aid the efficient profiling of the components (*125*). Brain lipids are often extracted and enriched using different ratios of organic solvents and water to aid the profiling process. Both untargeted and targeted MS approaches have been used for the profiling of brain lipids (*127*, *128*). Targeted quantitative profiling of a subset of lipids deemed potential biomarkers or involved in specific lipid pathways is mostly conducted using triple quadrupole (QQQ)–MS (*129*). While QQQ-MS systems are inappropriate for a comprehensive analysis of large lipidomes, the multiple reactions monitoring acquisition mode is extremely useful for the quantification of targeted lipid species associated with brain diseases (*130*). Conversely, untargeted lipidomic analysis is focused on extensively characterizing lipidomes of interest, requiring the use of high-resolution instruments such as quadrupole-TOF or Orbitraps. For example, Simons *et al.* (*125*) determined around 700 lipids from mouse brain tissues and cells using shotgun lipidomics and predicted the lipid pathways that are enriched in brain cells and in different regions. Shotgun MS, as well as LC-MS and GC-MS, have been used for the untargeted analysis of lipids. Recently, to enhance the confidence of lipid identification, ion mobility–MS, which provides high precision collision cross-sectional information, has been exploited to simultaneously explore numerous lipids obtained from brain cells and specific regions (*131*, *132*).

With advances in MS-based lipidomics, lipid databases (DBs) and software tools have been developed for monitoring large-scale datasets, for performing the compositional and structural profiling of numerous lipids, and for analysis of their molecular networks (Table 2). LIPID MAPS (Lipid Metabolites and Pathways Strategy) is well known as the largest lipid-only DB in the world and enables the structural annotation of approximately 40,000 lipid species (*133*). Now, the LIPID MAPS website not only serves as a platform for MS data interpretation but also allows the statistical analysis of biologically relevant lipids. Besides LIPID MAPS, lipidr and LipidSig are other lipidomic-centered DBs. Lipidr is a software tool package with open-source R/Bioconductor for data mining and analysis, and LipidSig is a user-friendly web server that enables the structural profiling of lipids, especially for FA-dependent analysis and network analysis with lipid-associated gene information (*134*, *135*). A summary of the DBs available for metabolomics, as well as solely for lipidomics, is provided in Table 2. The Metabolomics Workbench is the prominent portal for metabolites including lipids, most notably known as the data repository of the National Institutes of Health’s metabolomics (*136*). The entire collection of metabolites in this database amounts to approximately 60,000 species. The structural information for these species has been collected from LIPID MAPS, chemical entities of biological interest, the human metabolome database, the biological magnetic resonance bank, and the Kyoto Encyclopedia of Genes and Genomes. The Metabolomics Workbench allows the integration, analysis, and deposition of heterogeneous data produced from metabolomic studies using MS and NMR (*136*).

**Table 2.** Bioinformatic tools for lipidomics. KEGG, Kyoto Encyclopedia of Genes and Genomes. HMDB, human metabolome database; BMRB, biological magnetic resonance data bank.

Informatic tools*	**Datasets**	**Description**	**Access link**
LIPID MAPS	Lipidomics	• The largest lipid-only database covering 40,000 lipid species	www.lipidmaps.org
		• Data interpretation, structure annotation, and statistical analysis are available	
LipidSig	Lipidomics	• The web server capable of profiling and data mining of lipidomic datasets, exhibiting differential expression, correlation, and networks of lipid species	http://chenglab.cmu.edu.tw/lipidsig
lipidr	Lipidomics	• Open-source R/Bioconductor package for data processing and mining of lipidomic datasets	www.lipidr.org
Metabolomics Workbench	Metabolomics (including lipidomics)	• A portal for metabolomics data, well-known as the data depot of National Institutes of Health’s Metabolomics	www.metabolomicsworkbench.org
		• Information of around 60,000 metabolites is contained, which is collected from LIPID MAPS, HMDB, BMRB, and KEGG.	
MetaboLights	Metabolomics (including lipidomics)	• Database for metabolomics providing metabolite structures, their biological information, and reference spectra.	www.ebi.ac.uk/metabolights/studies
MetaboAnalyst	Metabolomics (including lipidomics)	• Platform for metabolomics providing raw MS spectra processing, comprehensive data normalization, statistical analysis, functional analysis, meta- analysis, and integrative analysis with other omics data.	www.metaboanalyst.ca
GNPS-massive	Metabolomics (including lipidomics)	• Web-based system to share raw, processed, or annotated tandem MS data.	https://gnps.ucsd.edu/ProteoSAFe/static/gnps-splash.jsp

*Databases and software tools

All these developments in MS-based analytical platforms and software tools for data interpretation have led to the availability of increasing reference data for lipidomic research, thereby facilitating large-scale studies. In recent years, these analytical tools and DBs have been used for brain lipidomics. However, many brain lipids are still unknown, increasing the need for their global and in-depth characterization.

### Integrated omics in brain diseases

Brain diseases, such as neurodegenerative diseases (e.g., AD and PD) and psychiatric diseases, commonly exhibit molecular and clinical heterogeneities, which makes the development of diagnosis and treatment strategies quite difficult. Multi-omics is a powerful and useful approach to unravel the molecular mechanisms of diseases (*137*). In particular, the integrative analysis of lipidomics and other omics, such as genomics, transcriptomics, proteomics, and metabolomics, helps reveal the alterations in lipid metabolism and lipid-mediated signaling associated with perturbations in cellular homeostases caused by environmental disturbances and pathological processes (*11*, *138*, *139*).

Table 3 indicates recent studies on brain diseases using integrative analyses of lipidomics with other omics. A previous study on AD has discovered biomarkers for the prediction of amyloid deposition using extensive molecular analyses through transcriptomics, metabolomics, and lipidomics (*137*). The biomolecules containing medium-chain FAs, 4-nitrophenol, and 64 transcripts were differentially quantified in blood samples of amyloid-positive and amyloid-negative patients, revealing the discriminant omics signature for predicting amyloid deposition. In another study on AD, Legido-Quigley *et al.* (*138*) conducted both lipidomic and proteomic analyses of plasma samples from normal subjects and patients with AD or mild cognitive impairment. They found that certain lipids involved in lipid metabolism and immune response and proteins that modified cytokine productions, plasma lipoprotein particle level regulation, and insulin-like growth factor receptor signaling correlate positively with brain atrophy and disease progression. This integrative omics approach has shown molecular networks of lipids and proteins associated with plasma lipoprotein particles and their correlation with AD phenotypes. A recent study on the integrative profiling of postmortem AD brain samples using transcriptomics and lipidomics have reported on the genetic mutation in the lipid carrier protein, APOE, caused by AD (*139*). Brain tissues genotyped as *APOE*ε*2*/carriers, *APOE* ε*3*/*3*, and *APOE*ε*4*/carriers have been used to determine changes in lipid patterns associated with mutations in the different *APOE* genotypes. The brain sample with *APOE* ε*4*/carriers exclusively revealed a decrease in specific lipids, such as nonbilayer-forming PE and PC and mitochondrial membrane–forming lipids. The correlation between altered lipid species and differentially expressed genes showed that differences in intracellular catabolic processes are associated with *APOE* allele–dependent differences in AD pathology. In the case of PD, the correlation between α-syn, genetic variants of the glucocerebrosidase (GCase) gene *GBA* (one of the genetic risk factors for PD), and lipid substrates of GCase, such as glucosylceramides, ceramides, and sphingosines, is explored using the CSF of patients with or without gene mutations (*140*). It has been found that patients with mutations in lysosomal GCase, displaying elevated levels of glucosylceramides and low levels of α-syn, exhibit a disturbance in lysosomal homeostasis. In addition to brain diseases, integrated omics has been used to understand the cellular and molecular mechanisms of brain region–specific functions and activities including demyelination (*141*, *142*).

**Table 3.** Integrated omics in brain diseases. MCI, mild cognitive impairment; TG, triglyceride; Cer, ceramide; ApoB, apolipoprotein B; PAFAH, platelet-activating factor acetylhydrolase.

**Brain disease**	**Model**	**Omics**	**Description**	**Reference**
AD	• Human	Transcriptomics, metabolomics, and lipidomics	• In the plasma samples of patients with or without amyloid deposit scanned by ^18^F-florbetapir PET	(*137*)
	• Blood		• (Amyloid, +) 64 genes involved in inflammation and FA metabolism were up-regulated (31) or down-regulated (33).	
	• Amyloid(+), *n* = 48/ Amyloid(−), *n* = 48		• Five metabolites (nonanoic acid, octanoic acid, undecanoic acid, hydroxy-nonanoic acid, and 4-nitrophenol) were inversely correlated with the amyloid burden.	
AD	• Human	Lipidomics and proteomics	• In the plasma samples of normal and patients with AD or MCI	(*138*)
	• Blood		• The lipid module composed of SMs, PCs, Cers, and TGs and the protein module containing ApoB, PAFAH, and P-cadherin were strongly correlated.	
	• AD, *n* = 185/MCI, *n* = 40/ Control, *n* = 185		• Both lipid and protein modules were linked to the regulation of plasma lipoprotein particle levels.	
AD	• Human	Lipidomics and transcriptomics	• In the brain tissues genotyped as *APOE*ε*2*/carriers, *APOE*ε*3/3*, and *APOE*ε*4*/carriers	(*139*)
	• Brain tissues		• (*APOE*ε*4*/carriers) the decrease of nonbilayer-forming PE and PC and mitochondrial membrane– forming lipids	
	• *APOE*ε*2*, *n* = 8/*APOE*ε*3*/*3*, *n* = 12/ *APOE*ε*4*, *n* = 22			
PD	• Human	Genomics, lipidomics, proteomics	• In the CSFs of patients with or without the variants in the glucocerebrosidase gene (*GBA*)	(*140*)
	• CSF		• (*GBA*) low glucocerebrosidase activity, high level of glucosylceramides, and low α-synuclein	
	• PD, *n* = 102/ Control, *n* = 414			

## CONCLUDING REMARKS

The brain, a lipid-rich organ, contains high levels of cholesterol, sphingolipids, and glycerophospholipids, due to the specific flux of lipids regulated by the BBB (*42*). These lipids, primarily involved in cell membrane formation, play critical roles in energy storage, the regulation of membrane fluidity and permeability, and signal transduction (*143*). Therefore, their dysregulation can cause brain diseases, such as neurodegenerative diseases and psychiatric diseases (*144*, *145*). Understanding the lipid metabolism in the brain, which is affected by physiological, pathological, and environmental conditions, can help untangle molecular pathologies of brain diseases. Brain lipidomics has been consistently investigated to discover potential biomarkers for the development of diagnostic and treatment approaches (*146*, *147*). Considering the molecular and clinical heterogeneities in brain diseases, integrated omics, including lipidomics, serves as a crucial method to identify accurate and robust biomarkers through the analysis of the cross-talk between the different biological systems (*148*).

In recent years, the characterization of brain lipids has progressed considerably thanks to advances in MS performance (*149*). The development of analytical platforms using high-resolution and accurate MS facilitates the comprehensive profiling of thousands of lipids and their structural analyses, which enables the enlargement of existing lipidomic DBs. These advancements in analytical tools and DBs enable the identification of unknown lipid species in the brain and aid in the deposition and, therefore, the underpinning of molecular information for determining the extensive alterations in lipid species and the associated biological systems, caused by brain diseases.

**Fig. 3.**
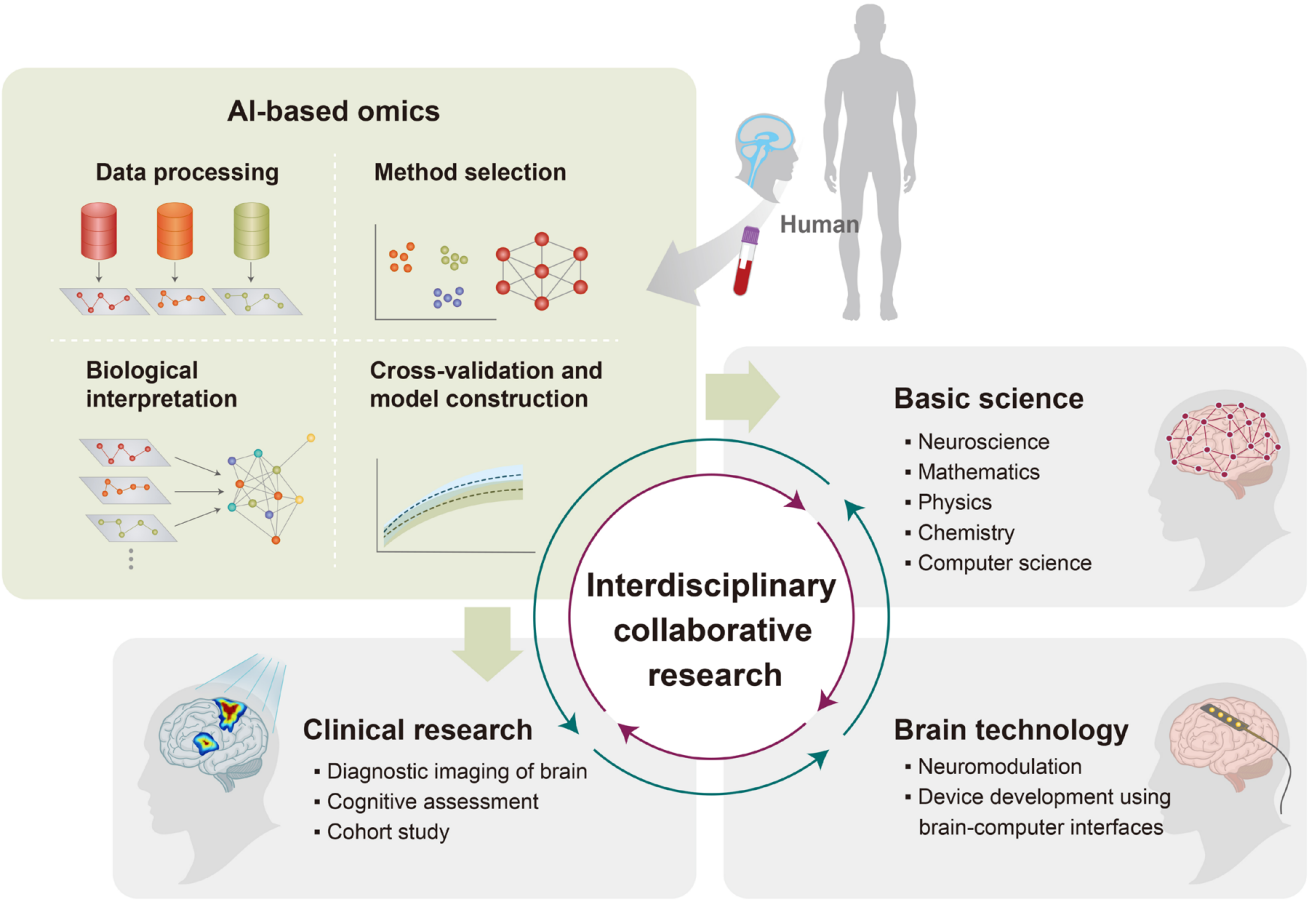
Future direction for brain research using multi-omics. The advancement of brain research requires interdisciplinary collaborative research encompassing fields such as basic science, technology, and clinical research, to systematically understand brain functions and medical applications. As a starting point, the analysis of human brain specimens using multi-omics coupled with AI technology will provide critical information necessary to untangle the molecular networks in the brain. Lipidomics serves as the main component of brain omics.

MS is the gold standard for generating high-throughput data for omics research, especially in proteomics, metabolomics, and lipidomics (*13*, *150*, *151*). With the rapid generation of large amounts of data by MS, the interpretation of vast amounts of biological data has turned into a colossal challenge. In recent years, artificial intelligence (AI)–based bioinformatics has emerged as a suitable tool for extracting meaningful information from complex and multidimensional datasets (Fig. 3) (*143*). In brain diseases exhibiting complex pathogenesis, machine learning allows the integration of lipidomics, other omics, and clinical data, thereby revealing molecular networks associated with different pathological phenotypes. Lipidomics in brain research plays a key role in bridging the gap between the phenotype and genotype associated with the disease, as it reflects variations in the genome, transcriptome, and proteome. Therefore, combining brain omics research with AI-based platforms will facilitate large-scale cohort studies and long-term follow-ups, enabling preventive medicines for brain diseases with high heterogeneity in terms of biological changes and spatial-temporal progression.

The fundamental agenda in brain research involves understanding the structural and functional characteristics of molecular organization in the brain, starting from the genes to the whole organ, and the development of treatment approaches for brain diseases and psychiatric diseases. To achieve this goal, the use of human resources is inevitable because animal models used often demonstrate limitations in reflecting the physiological state of humans owing to the differences in the genotype and life cycle. In addition, interdisciplinary collaborative research between different fields such as neuroscience, mathematics, physics, computer science, engineering, and medicine is required for the systematic investigation of brain functioning and the practical application of research results (Fig. 3). Unlike other organs in the human body, the brain has diverse functional abilities that are affected by intricate neural circuits. To comprehend cellular mechanisms and intercellular communications necessary for brain functioning, it is essential to perform in-depth and extensive molecular studies on the brain, at multiple levels. Large-scale genomic, transcriptomic, and proteomic analyses of the brain have been extensively explored to understand brain pathology. In addition, integrative analyses involving these omics aid in revealing alterations in the central dogma. Deep insight into metabolomic and lipidomic analyses (the omics most closely correlated with phenotypes) is essential to comprehend the occurrence and progression of brain diseases. Although the study on brain lipidomics is still in its infancy worldwide, we believe that vast amounts of meaningful data obtained through lipidomics will serve as a valuable resource for further data-driven clinical research on the brain.
