# Supplementary material for: Brain lipidomics: From functional landscape to clinical significance
Source: Sci Adv. 2022 Sep 16;8(37):eadc9317. doi: 10.1126/sciadv.adc9317 (PMC9481132; doi:10.1126/sciadv.adc9317)

Science Advances
AAAS

Supplementary Materials for

Brain lipidomics: From functional landscape to clinical significance

Jong Hyuk Yoon *et al.*

Corresponding author: Jong Hyuk Yoon, jhyoon@kbri.re.kr

Sci. Adv. **8**, eadc9317 (2022)
DOI: 10.1126/sciadv.adc9317

This PDF file includes:

Fig. S1

Fig. S1. Sphingolipid pathway

The pathway contains representative sphingolipids found in the brain. Ceramide is a common biosynthetic precursor of all glycosphingolipids, sphingomyelins, sulfatides, and cerebrosides. The sphingolipid metabolic enzymes are indicated in the synthetic paths.

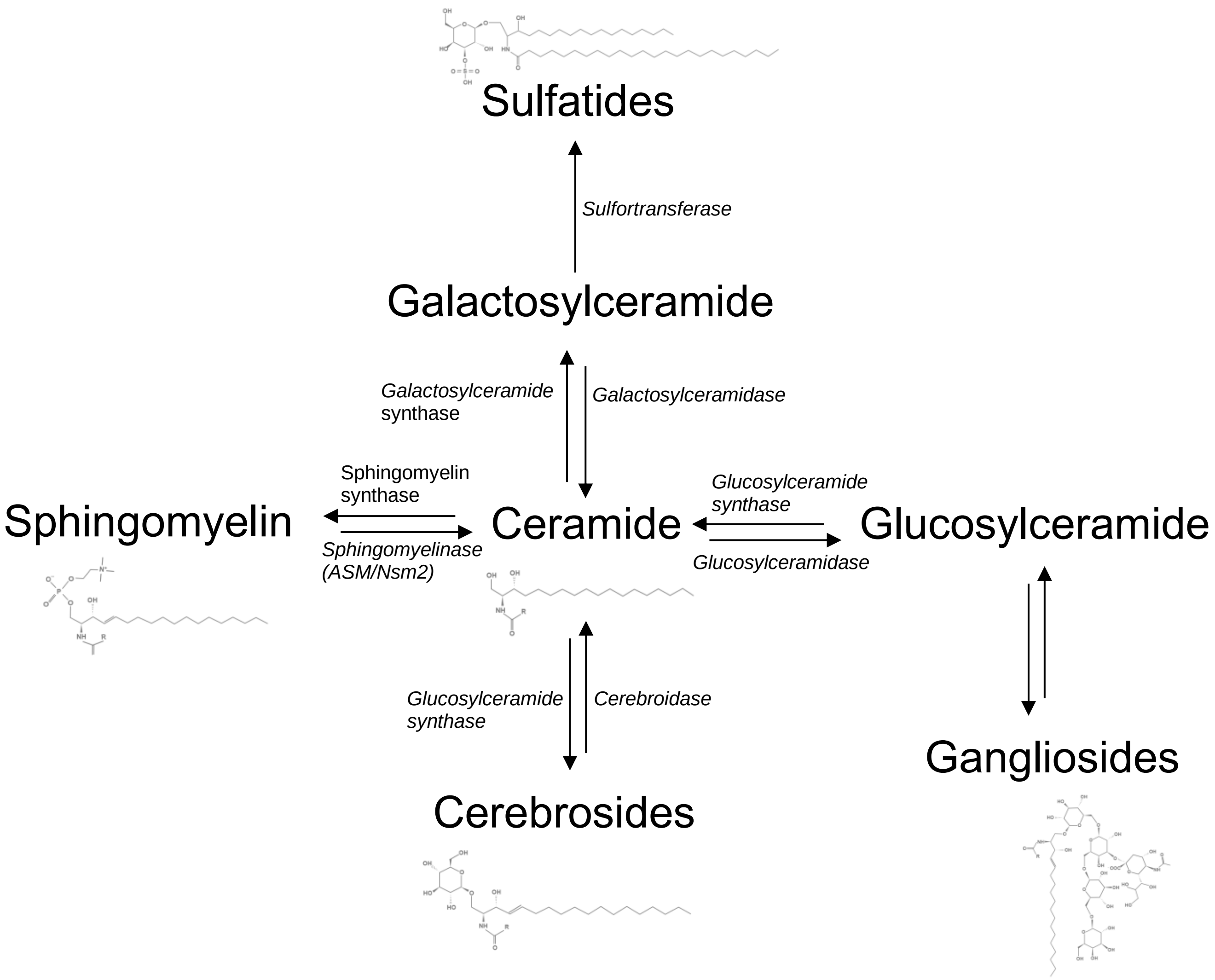

Supplement: Supplementary file 1 — Fig. S1 [file sciadv.adc9317_sm.pdf]
